# The role of 4-dimensional flow in the assessment of bicuspid aortic valve and its valvulo-aortopathies

**DOI:** 10.1259/bjr.20220123

**Published:** 2022-07-29

**Authors:** Caryl Elizabeth Richards, Alex E Parker, Aseel Alfuhied, Gerry P McCann, Anvesha Singh

**Affiliations:** 1 Department of Cardiovascular Sciences, University of Leicester and the National Institute for Health Research Leicester Biomedical Research Centre, Glenfield Hospital, Leicester, UK; 2 Leicester Medical School, University of Leicester, Leicester, UK

## Abstract

Bicuspid aortic valve is the most common congenital cardiac malformation and the leading cause of aortopathy and aortic stenosis in younger patients. Aortic wall remodelling secondary to altered haemodynamic flow patterns, changes in peak velocity, and wall shear stress may be implicated in the development of aortopathy in the presence of bicuspid aortic valve and dysfunction. Assessment of these parameters as potential predictors of disease severity and progression is thus desirable. The anatomic and functional information acquired from 4D flow MRI can allow simultaneous visualisation and quantification of the pathological geometric and haemodynamic changes of the aorta. We review the current clinical utility of haemodynamic quantities including velocity, wall sheer stress and energy losses, as well as visual descriptors such as vorticity and helicity, and flow direction in assessing the aortic valve and associated aortopathies.

## Introduction

Bicuspid aortic valve (BAV) is the most common congenital cardiac malformation, affecting 1–2% of the population.^1,2^ The significant heterogeneity in its valvular and aortic phenotypes, clinical presentations, associated disorders and prognosis present multifaceted challenges.^3,4^ Recent international consensus recognises three BAV types: fused BAV, two-sinus BAV and partial-fusion BAV, each with specific phenotypes.^5^ Fused BAV is the most common type, defined by fusion of two of the three cusps (Type 1 has fusion of the right and left coronary cusps (RL-BAV), Type 2 has right and non-coronary fusion (RN-BAV) and Type 3 has left and non-coronary fusion (LN-BAV).^6^

Patients with BAV are likely to develop aortic valve dysfunction, such as aortic stenosis (AS) or regurgitation, at a much younger age than those with tri-leaflet aortic valves (TAV). BAV is also associated with an increased incidence of aortopathies including larger dimensions of the proximal aorta and increased incidence of aortic aneurysms and dissection.^7^ Coarctation of the aorta is also commonly associated with BAV.^8^

The aetiology of bicuspid aortopathy is still debated but two theories prevail. The first is that a congenital disorder of vascular connective tissue increases the aortic wall fragility, which is therefore prone to damage. Alternatively, according to the haemodynamic theory, altered turbulent flow patterns, changes in peak velocity and wall shear stress (WSS) are associated with aortic wall remodelling. In reality, both theories are thought to coexist.^9^

Transthoracic echocardiography is the current gold-standard for diagnosis and evaluation of AS severity and relies on Doppler assessment of flow across the aortic valve.^10^ However, the heterogeneity in aortic geometry and blood flow parameters, even among AS patients presenting with similar AS severity, suggests a need to broaden the classification system. Time-resolved 3D phase-contrast MRI (3D PC MRI), or 4D flow MRI, enables the non-invasive, *in-vivo* study of vessel anatomy and complex blood flow patterns. Anatomic and functional information acquired from 4D flow images combine the advantages of cardiac MRI with echocardiography, and can illustrate pathological geometric and haemodynamic alterations of the thoracic aorta, including assessment of the aortic valve function.

We review the basic principles of 4D flow MRI and evaluate the current status of the resulting haemodynamic biomarkers as predictors for improving risk stratification and intervention timing in patients with BAV-associated aortopathies.

## 4D flow magnetic resonance imaging principles

### 4D flow data acquisition

PC MRI is achieved with bipolar velocity encoding gradients applied in the slice-selection direction, orthogonal to the imaging plane slice (“through-plane” encoding).^11^ Phase shifts induced in protons (herein referred to as spins) in stationary tissues are nullified by the opposing bipolar gradient. However, spins that have moved along the applied gradient will retain their phase that is directly related to their velocity. The resultant phase difference is isolated to the moving spins and is proportional to their through-plane velocity.^12^

For cardiovascular applications, integration of 2D PC into the MRI protocol has already enabled 2D velocity mapping in a region-of-interest in the imaging plane perpendicular to the vessel of interest.^13^ In a typical 2D PC MRI protocol, an oblique plane selected perpendicular to the ascending aorta (AAo) at the level of the aortic valve permits transvalvular assessment of peak velocities, pressure gradient and retrograde flow (or regurgitant fraction) similar to Doppler echocardiography for measuring AS severity.^14^

Full 3D cine enables retrospective visualisation and quantification in the entire 3D volume in any desired plane without the restrictions of a predefined imaging plane as in 2D PC MRI. For aortic imaging, 4D flow is most effectively acquired with a sagittal oblique volume covering the thoracic aorta, including the left ventricular outflow tract, AAo, aortic arch and descending aorta. Further reductions to acquisition times of the order of 5–10 min are achieved by combining interleaved 4-point velocity encoding with k-space segmentation.^11^

In k-space segmentation, multiple lines of k-space are acquired per segment after each trigger.^15^ This decreases the imaging time by a factor equivalent to the number of segments and permits simultaneous acquisition of multiple cardiac phases or anatomical slices. Navigator gating of the diaphragm motion at the end of each cardiac cycle aids respiration control and minimises breathing artefacts.^16^ Using the 4-point technique, four simultaneous velocity-encoded acquisitions in all three x-, y-, z-directions are acquired for each single k-space line.^11^ Other k-space acquisition trajectories (*e.g.* echo planar, spiral, and radial) can also accelerate acquisition speed but can be challenging to implement and reconstruct in post-processing.^17–19^

**Figure 1.**
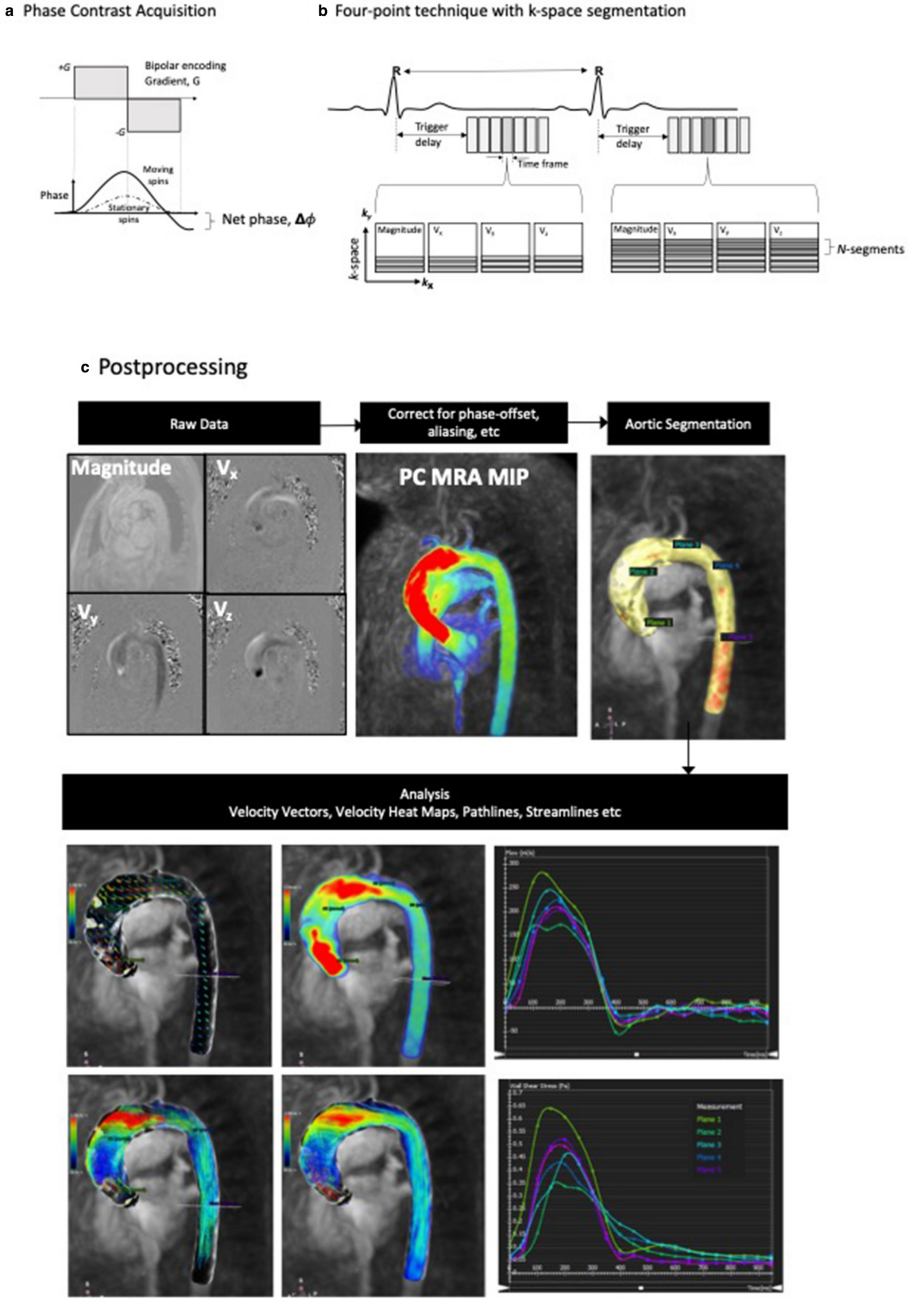
A. Typical 4D flow data acquisition of the thoracic aorta. (A) In the Cartesian system, bipolar velocity encoding gradients are applied in the orthogonal x-, y-, and z-directions. Phase shifts induced in stationary tissues are nullified by the opposing bipolar gradient. The net phase shifts, ΔΦ, are proportional to the velocity, ν, of the moving spins in each direction. (B) In the 4-point technique, four simultaneous acquisitions comprising the anatomic reference data and three blood-flow velocity encoded data sets along the ν_x_, ν_y_, and ν_z_ directions are acquired for each time frame in the R-R interval. In k-space segmentation, multiple lines of k-space are acquired per segment after each trigger. (C) Phase-offset and aliasing corrections are applied to the raw images and the PC MRA generated. Surface rendering with a user-defined threshold (CVI42 Circle) and manual adjustment can semi-automatically segment the thoracic aorta for visualisation of time-resolved velocities, pathlines and streamlines and further analysis. PC MRA, phase-contrast MR angiography.

During each heartbeat, the R-R interval of the cardiac cycle is divided into multiple time-resolved cine frames. Cine images are acquired by repeat imaging over multiple cardiac cycles using electrocardiogram-gated synchronisation. Image reconstruction yields a series of magnitude (anatomical) and phase-difference (velocity-encoded) images that track the temporal evolution of the anatomy and velocity on the imaging plane slice respectively. Figure 1 shows a typical 4D flow acquisition and magnitude and velocity-encoded images of the thoracic aorta.

### 4D flow sequence parameters

Radiofrequency-spoiled gradient echo sequences with the minimum achievable echo and repetition times, TE and TR, are typically used to saturate background and stationary tissues.^17^

Optimal velocity sensitivity relies on a user-defined velocity encoding parameter, Venc, that corresponds to a phase shift of ± π (±180 degrees). Compromise is required between high Venc to avoid aliasing in the velocity images and low Venc to reduce noise and improve image quality. Changes in Venc also correspond to changing the strengths and duration of the velocity encoding gradient, thus influencing the minimum achievable TE and TR. Typical velocity sensitivities are Venc = 150 cm/s for aortic flow measurements and Venc = 100 cm/s for flow in the pulmonary artery, with higher velocity sensitivities in the presence of AS.^13^

Compromise is also required between spatial resolution, scan time and signal-to-noise ratio. Spatial resolution should be as high as possible and temporal resolution should be as short as possible for accurate flow quantification and to identify smaller-scaled temporal variations in pulsating blood flow. However, the smaller the voxel and the greater the number of temporal steps per cycle, the longer the scan time and the lower the signal-to-noise ratio. In 4D flow, voxel sizes of 2.5–3.0 mm and a temporal resolution of around 40 ms are typical for pulsatile aortic flow measurements.^17,20,21^ Sources of error such as velocity aliasing and background phase contributions from eddy-currents, Maxwell terms, and gradient field distortions, that are not completely eliminated by the initial subtraction operation, require post-processing correction strategies.^22–24^

## Utilising aortic 4D flow MRI in aortic valve disease

**Figure 2.**
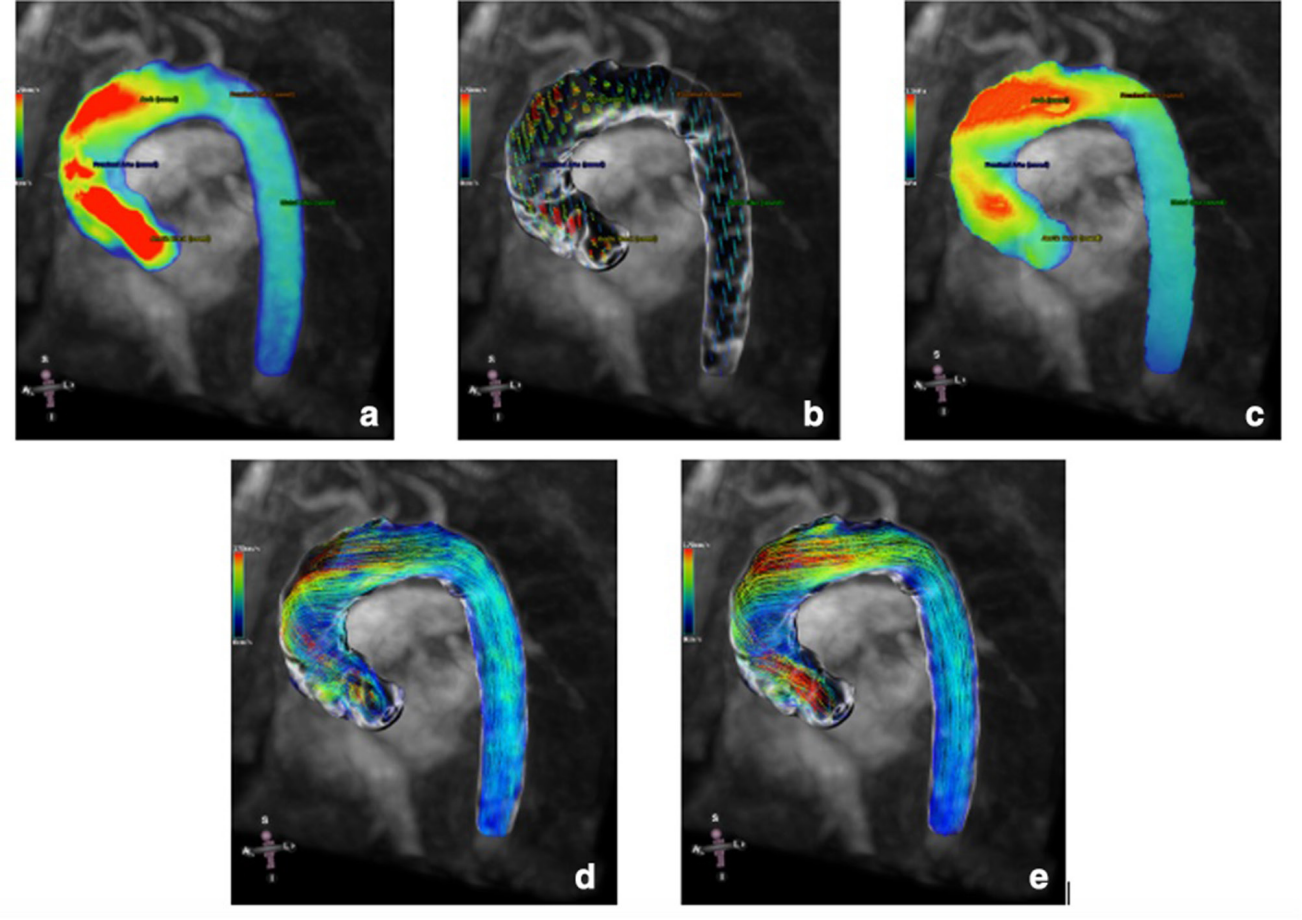
An example of the blood flow visualisation from volumetric images of a thoracic aorta using 4D flow in a patient with bicuspid aortic valve and severe aortic stenosis confirmed by echocardiography. Images were generated with commercial software, CVI42 (Circle Cardiovascular Imaging Inc., Calgary, Canada) depicting: (A) velocity heat map, (**B**) resultant velocity vectors overlaid with the velocity heat map, (**C**) maximum wall shear stress heat map, (**D**)pathlines, and (E) streamlines, all derived at a single timeframe in the cardiac cycle. Areas of maximum wall shear stress (indicated by the red areas) are shown to match the high peak velocities with a pronounced helical flow pattern.

Commercial software permits surface rendering of the vascular structures of interest to generate a non-contrast PC MRI angiogram for further analysis and visualisation of 3D blood-flow pattern, shown in Figure 2. Streamlines represent the instantaneous tangent to the blood flow velocity vector for a single cardiac timeframe. Pathlines are the trajectories that individual blood fluid elements follow over the cardiac cycle. The direction the path takes is determined by the streamlines of the fluid at each moment in time.^25^ “Forward flow” refers to the movement of blood in the expected direction of movement while “reverse flow” is equivalent to regurgitant volume in the opposite direction. “Net flow” is the sum of these two figures.

Aortic flow angle represents the angle between the line perpendicular to the short axis analysis plane of the aorta and the instantaneous mean flow vector at peak systole.^26^ Flow displacement is defined as the distance between the centre of the lumen and the centre of the forward velocity-weighted centre of mass position, and normalised to the lumen diameter.^27^ Elevation of these values are an indicator of outflow asymmetry and eccentricity.^27–30^

4D flow also allows quantification of advanced haemodynamic biomarkers using the 3D velocity values per voxel. These including WSS, vorticity, helicity, turbulent kinetic energy, viscous energy loss, pressure mapping and pulse wave velocity (PWV), summarised in Table 1. Since velocity is measured at finite, discrete points, several numerical schemes have been applied to approximate the differential values with varying levels of accuracy and signal-to-noise ratio.^33^ The challenge with 4D flow-derived parameters is to find a numerical method that accurately characterises these flow structures and links them to abnormal valve morphology within the available spatial and temporal resolution.

**Table 1.** Summary of advanced haemodynamic metrics and measurements derived from 4DFlow MRI

Metric	Formula	Description
Jet angle		The angle between the mean velocity vector and the normal to the cross-sectional plane.
Flow displacement	$FlowDisplacement=\frac{Distance(C_{vel},AoC)}{AoDiameter}$ Where $C_{vel,j}=\frac{\sum_{i}r_{i,j}\left|\nu_{i}\right|}{\sum_{i}\left|\nu_{i}\right|}$	The distance between the vessel centreline (AoC) and the centre of the eccentric flow (C_vel_) and is normalised for overall vessel size. *i* = lumen pixel *j* = *x, y, z*
WSS	$\tau =\mu {\left.\frac{\partial \nu }{\partial n}\right|}_{wall}$	The drag force exerted by the vessel endothelium on the blood flow. Abnormal levels of WSS are associated with cardiovascular pathologies.^31^ *μ* is the dynamic viscosity ν is the velocity *n* is the local wall normal displacement vector
Vorticity	$\overset{-}{\omega }=\nabla \times \overset{-}{\nu }$	Describes the revolving, circular motion, around an axis without any forward motion.
Helicity	$\overset{-}{\nu ∙\omega }$	Helical flow occurs when particles rotate around an axis of flow whilst also exhibiting a forward motion.
Turbulent kinetic energy	$\frac{1}{2}\rho \sum_{j=1}^{3}\sigma_{j}^{2}$	Turbulent kinetic energy measures the dynamic losses due to random spatial and temporal variation in fluid velocity at the microscopic scale that eventually dissipate into thermal energy.^32^ ρ is the fluid density σ is the variance of the velocity fluctuations, assuming a Gaussian velocity distribution within the voxels *j* = *x, y, z*
Viscous energy loss	$\emptyset_{\nu }=\frac{1}{2}\sum_{i}\sum_{j}{\left[\left(\frac{\partial \nu_{i}}{\partial x_{j}}+\frac{\partial \nu_{j}}{\partial x_{i}}-\frac{2}{3}\left(\nabla ∙\nu \right)\delta_{ij}\right)\right]}^{2}$ δ_ij_ = 1 for *i* = *j* and δ_ij_ = 0 for *i ≠ j*	Viscous energy loss corresponds to the irreversible process by which the work done by a fluid on adjacent layers due to the action of shear forces is transformed into heat induced by fluid viscosity and *no‐slip* condition. Based on a reformulation of the viscous portion of the Navier–Stokes energy equations.
PWV	$\frac{∆d}{∆t}$ Bramwell-Hill equation $\frac{1}{\sqrt{\rho \cdot D}}$	Rate at which a pressure wave generated at systole moves along the arterial tree. It is the clinical standard for assessing aortic stiffness. Δ*d* is the distance travelled by the pulse wave divided by the time taken, Δ*t*, for the wave to travel this distance. PWV is inversely related to distensibility by the Bramwell-Hill equation, assuming the vessel wall thickness is small compared to the diameter. ρ is incompressible fluid density *D* is distensibility of a compressible tube
Pressure mapping	$-\nabla p=\rho \left(\frac{\partial \nu }{\partial t}-\nu ∙\nabla \nu -g\right)-\mu \nabla^{2}\nu$	Pressure gradient ▽*p* is computed from the flow velocity field using momentum conservation of the Navier–Stokes equation, and assumes blood to be an incompressible laminar Newtonian fluid Applications are challenging in cases of turbulent stenotic flow. *ρ* is incompressible fluid density *µ* is dynamic viscosity *g* is gravitational force vector

PWV, pulse wave velocity; WSS, wall shear stress.

While flow phenomena in the aorta can be appreciated visually using the path- and streamlines, evaluation remains limited by the observers’ subjective assessments. Qualitative and semi-quantitative approaches have demonstrated potential correlations between BAV and TAV aortic valve disease and these advanced haemodynamic parameters.^25^ However, these studies are either feasibility studies, or are typically small in size and highly dependent on the analysis method developed. The main findings of studies utilising 4D flow in aortic valve disease are summarised in Table 2, which also includes a breakdown of the cohorts and study limitations. These exemplify the mixture of BAV patients either with and without AS and/or aortic dilation and non-uniform “control” groups, including TAV volunteers with and without aortic dilatation, with and without gender- and age-matching. The heterogeneity in both patient and control subgroup populations further limits between-study assessment of the accuracy and validity of these parameters, especially for establishing physiologic normal values.^48^

**Table 2.** Summary of studies using 4D flow MRI of the aorta in BAV and TAV subjects

Authors	Sample size	Disease	4D flow parameters	Summary	Limitations
Guala^34^	47 BAV (37 RL-BAV; 10 RN-BAV).	70.2% aortic dilatation.	WSS	Both WSS (magnitude) and WSS (circumferential were independently and statistically significantly associated with local growth rate at the level of the pulmonary artery bifurcation.	WSS maps using manually located landmarks. Semi-automatic assessment of aortic diameters and growth rates between two time frames using CT angiograms.
Minderhoud^35^	32 BAV (8 type 0; 16 RL-BAV; 4 RN-BAV; 4 Type 2), 28 TAV		WSS	Significant volumetric growth of the proximal AAo occurred in BAV patients. Only WSS (angle) independently associated with proximal aortic growth. WSS (angle) and WSS (magnitude) independently associated with growth in entire AAo.	Volumetric growth measured by CT angiograms may result in inconsistencies. Small sample size with no BAV subtype.
Soulat^36^	72 BAV, 136 TAV	None-to-moderate AS and AR severity	WSS	Greater area with elevated WSS in BAV patients with faster AAo growth rates.	Growth rates split into faster and slower AAo growth rate groups based on arbitrary mean threshold.
Lenz^37^	20 (15 BAV, 5 UAV)	Symptomatic AR	Peak velocity; forward flow; reverse flow; helical flow; vortical flow; flow displacement; WSS	Assessment of changes in blood flow dynamics in AVR. Significant reduction of AR. Helical and vortical flow, flow displacement and WSS were reduced post-surgery.	Manual positioning of 2D analysis planes. Male gender bias. Small sample size.
Dux-Santoy^38^	46 BAV, 44 controls	Mild to moderate AS (maximum velocity 3 m/s)	Peak systolic WSS; OSI	Increased WSS and decreased OSI in BAV compared to healthy volunteers. Regions with low WSS and high OSI did not match those with the greatest dilation suggesting these two parameters implicated in aortic dilation.	Only patients with non-severe valvular dysfunction and relatively normal aortic diameters were included.
**Fatehi Hassanabad** (**2020**)^39^	32 BAV, 11 controls	Varying degrees of aortic stenosis and regurgitation	Pressure drop at nine predefined levels from LVOT to distal descending aorta	Pressure drop between LVOT and other levels were significantly more increased in BAV patients than healthy volunteers	Small sample size. Analyst not blinded from patient or volunteer during analysis. Limitations from discrete spatial and temporal resolution.
Dux-Santoy^40^	111 BAV, 21 TAV, 24 Healthy controls	Non-severe valvular disease	Peak velocity; jet angle; displacement; in-plane rotational flow, WSS; systolic flow reversal ratio	Increased eccentricity and rotational flow observed in BAV proximal aortic arches compared to TAV. Arch dilation independently associated with RN-BAV, rotational flow and systolic flow reversal ratio. Suggested that increased rotational flow explained dilated proximal arch in male BAV patients with valvular stenosis.	Limited number of analysis planes. WSS is thought to be underestimated.
**Guala** (**2019**)^41^	234, 136 BAV	No significant valvular disease	PWVs; aortic distensibility	Investigated aortic stiffness of BAV patients compared to dilated AAo TAV or Marfan’s Syndrome. Non-dilated BAV patients had similar PWV and aortic dilation to the healthy controls. AAo PWV showed biphasic pattern in BAV patients. AAo and DAo stiffness in Marfan’s were greater than BAV. PWV was independently associated with AAo dilation in BAV patients.	Age matching was not considered. Only Caucasian patients examined.
Farag^42^	14 TAVR, 14 SAVR	Study conducted post-op. Procedures were for symptomatic AS.	Velocity; WSS; eccentricity; flow displacement	TAVR was associated with blood flow eccentricity and displacement in both the mid and distal AAo, whereas SAVR changes only occurred in the distal AAo. TAVR group also had increased blood flow velocity and WSS in AAo.	Small sample size. Imaging was not conducted prior to surgery to allow for comparison.
**Rodríguez-** Palomares^43^	101 BAV, 20 healthy subjects	No severe valvular disease	Peak velocity; flow jet angle; normalised flow displacement; in-plane rotational flow; systolic flow reversal ratio	RL-BAV demonstrated anterior distribution of flow. RN-BAV had a posterior outflow jet which moved to anterior or right anterior in the mid AAo. Velocity profiles for both BAV groups matched the location of maximum systolic axial WSS. RN-BAV had higher rotational flows in all levels of the aorta.	WSS estimations were only conducted in 8 2D planes. Volumetric WSS would allow more in-depth analysis. Measurements may be underestimated.
Farag^44^	48 BAV, 25 TAV controls	Of BAV patients – 10 had AS (4 mild, 4 moderate and 2 severe). 2 patients with AS also had AR. 10 additional patients had AR	Peak systolic velocity; WSS	15% of surface area of AAo in BAV patients with AS had elevated WSS, in comparison to 6% non-AS BAV patients. Extent of increased WSS was concluded to be highest in those with AS and a nondilated AA.	Heterogeneity and small sample size. No individuals above age 65 included.
Binter^45^	51 (11 BAV)	Of the BAV patients 27 has severe AS, 24 had mild/ moderate	Velocity; Turbulent Kinetic Energy	Mean pressure gradient and turbulent kinetic energy weakly correlated. Dilated AA had significantly elevated turbulent kinetic energy but mean pressure gradient was lower. BAV patients showed increased turbulent kinetic energy. Confirmed additional features from 4D Flow compared to echocardiography.	Viscous losses were not evaluated in this study. Velocity gradients at the vessel wall were also excluded.
**Von Knobelsdorff-Brekenhoff (2016**)^46^	37 with AS (16 BAV), 37 Healthy controls	Of those with AS – 14 were graded as mild, 8 as moderate and 15 were severe	Helical flow; vortical flow; peak velocity flow; WSS.	AS patients with BAV and TAV had marked helical and vortical flow in AAo compared to controls.	Helical and vortical flow patterns were only assessed qualitatively. No BAV in healthy volunteers.
Lorenz^47^	16 BAV, 12 Healthy	Of BAV patients – 6 AS, 2AS+dilated AAo, 4r with dilated AAo and 4 normal	Time-resolved relative helicity	BAV patients demonstrated increased relative helicity *vs* controls. Retrograde flow in diastole. Global increase in helicity in the AAo during systole and DAo during diastole.	Resolution of aortic cross-sections meant small local helical flows may be undetected. Averages taken in analysis. No age-matched control group. Large VENC resulted in poor signal-to-noise.
Bissell^26^	142 (95 BAV, 47 healthy)	24 BAV patients had AS, 71 did not; no classification of severity	VENC; helicity; WSS; systolic flow angle	BAV patients had predominantly right-handed helical flow in AAo and larger aortas, higher helical flow and WSS. RN-BAV patients showed more severe abnormalities than RL-BAV. Similar aortic distensibility, aortic strain and PWV across all groups.	WSS measurements may be underestimated due to low resolution. Cross-sectional design.
**Mahadevia (2013**)^29^	75 (30 BAV, 30 TAV, 15 Controls)	Majority of patients had mild AS or regurgitation but none exceeded moderate disease	Systolic valve flow angle; outflow asymmetry; regional WSS.	Authors’ own aortopathy classification. Most BAV patients were Type 2 (enlargement of the tubular portion of AAo). Healthy patients demonstrated uniform velocity distributions. BAV patients had eccentric outflow jet patterns. Flow impacted the right anterior wall for RL-BAV, but the right posterior wall for RN-BAV. Flow displacement was the most sensitive parameter to differences in BAV phenotype.	Age differences in match controls. Recruitment strategy unclear.

BAV, bicuspid aortic valve; OSI, oscillatory shear index; SAVR, surgical aortic valve replacement; TAV, tri-leaflet aortic valve; TAVR, transcatheter aortic valve replacement;WSS, wall shear stress.

### Aortic valve velocity and flow direction

AS severity is defined by the aortic valve area and the transvalvular pressure gradient derived from the flow velocity using the continuity equation.^49^ The smaller the aortic valve area, the higher the peak transvalvular flow velocity.^44^ Peak flow velocity across a normal aortic valve is approximately 1.0 m s^−1^, which increases to 2.5–2.9 m s^−1^ and 3.0–4.0 m s^−1^ in mild and moderate stenosis respectively. If severe disease is present, forward flow may be greater than 4.0 m s^−1^.^50^ 4D flow has demonstrated significantly higher peak systolic velocities in both BAV and TAV patients in the presence of AS, although these may be underestimated compared with transthoracic echocardiography and 2D PC MRI values.^14,51^

Aortic regurgitation has also been measured in a subgroup of BAV AS patients.^37,52,53^ A statistically significant decrease in average systolic peak velocity was demonstrated in patients with symptomatic aortic regurgitation (15 BAV and 5 unicuspid aortic valves) after successful aortic valve repair, suggesting that the improved aortic valve geometry resulted in a reduction in forward-, reverse-, and net-flow.^54^ A reduction in the mean aortic diameters at the levels of the anulus, bulbus and mid AAo was also noted, although the additional aortic remodelling from suture annuloplasty or Dacron prostheses in some patients likely influenced this. The study was also limited by male gender bias in the cohort that may have resulted in an overestimation of the measurements given their increased heart and major blood vessels sizes,^55^ and the error arising from manual positioning of the 2D analysis planes.^56^

### Aortic helicity and vorticity

**Figure 3.**
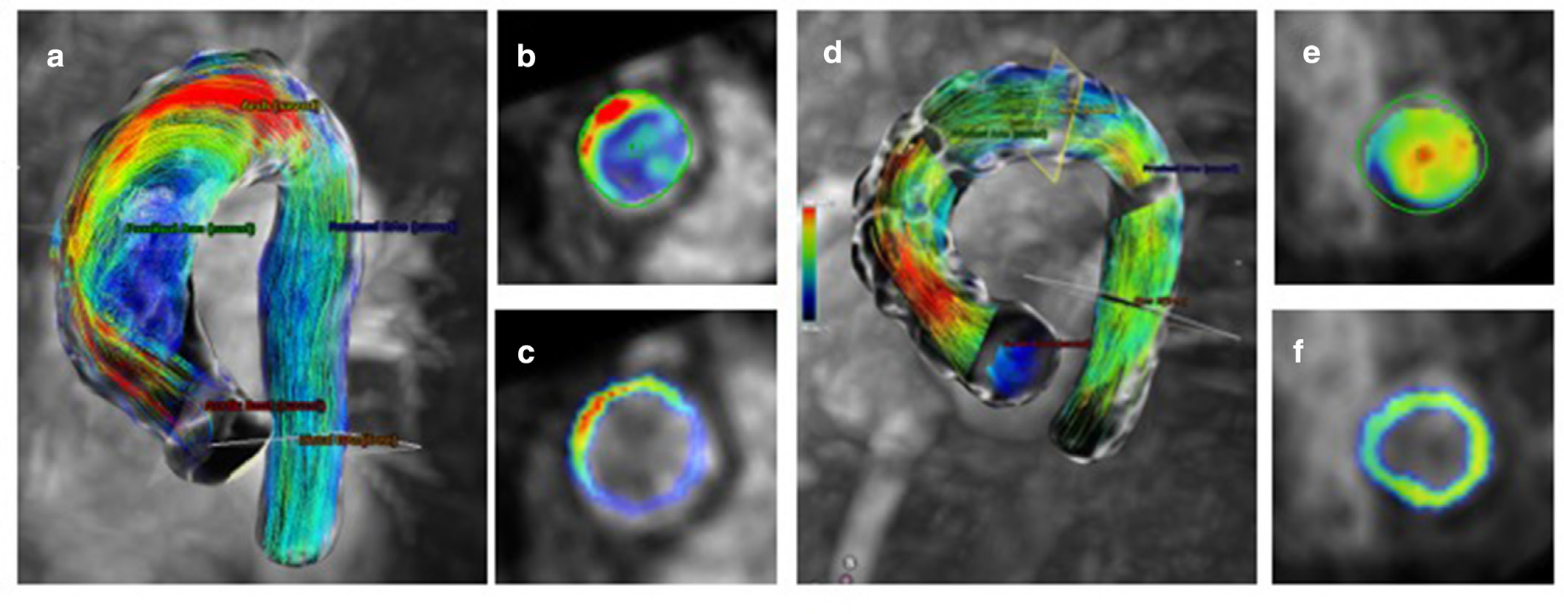
4D flow dynamics at peak systole in a 76-year-old male with tricuspid aortic valve and symptomatic aortic stenosis (NYHA class 2) using CVI42 (Circle Cardiovascular Imaging Inc., Calgary, Canada) demonstrating (**A**) helical flow with maximum axial velocity (red and yellow areas) moving outwards from helix centre resulting in (**B**) eccentric flow and (**C**) axisymmetric wall shear stress. Corresponding flow dynamics at peak systole in a healthy volunteer with tricuspid aortic valve demonstrating (**D**) dominant laminar flow with (**E**) uniform velocity distribution at the mid-ascending aorta perpendicular plane resulting in (**F**) symmetric wall shear stress.

Helical flow is a feature of both normal and abnormal aortas arising from the curved geometry of the thoracic aorta and left ventricular twisting during systole.^57–59^ Physiological helicity is thought to stabilise flow, preserve energy, and reduce flow stagnation offering some protection from atherosclerosis.^47,60^ Pathologically altered helicity and vortical flow formation in the presence of valve disease exert detrimental effects. These include geometrical changes due to abnormal mechanical wall stresses thought to lead to the incidence of aortic dilation in BAV.^61^ 4D flow can provide a detailed evaluation of the spatial and temporal distribution of helical and vortical flow in BAV aortopathies,^32^ with moderate to high inter- and intraobserver agreement depending on the method used.^31,62^ Figure 3 illustrates increased helical and eccentric blood flow resulting in axisymmetric WSS in the AAo of a 76-year-old patient with symptomatic severe AS compared to the laminar concentric flow in a healthy volunteer.

In a study comprising 20 patients with symptomatic aortic regurgitation, 4D flow imaging demonstrated right-handed helical flow in the AAo of all patients and vortical flow patterns in the AAo of 15 patients prior to aortic valve repair.^54^ A significant post-surgical reduction in pathological, secondary helical flows was recorded in 13 patients and vortex formation was reduced to 9 patients, attributed to an improvement in valve symmetry and competence. Increasing the median time from 9 days between pre- and post-aortic valve replacement scans may have allowed for further potential remodelling resulting in a change in helical flow in more patients.

Strong negative correlation was demonstrated between the aortic valve area and vortical flow, eccentricity and flow displacement in the AAO of patients with mild to severe AS (16 BAV and 21 TAV) compared to healthy controls.^63^ Vortical flow formations were also noted to be more pronounced in BAVs than TAVs, but there was a lack of further exploration into BAV subtypes and its resulting impact on blood flow. In a larger study (120 BAV, 24 healthy volunteers and 21 TAV with proximal aortic arch dilatation), flow dynamics and dilation in the aortic arch were investigated for each BAV fusion type.^40^ Increased eccentricity and rotational flow were demonstrated in the proximal aortic arches of BAV patients compared with TAV. RN-BAV, rotational flow and flow reversal ratio were also found to be independently associated with arch dilation. The authors hypothesised that the increased rotational flow in BAV explained the dilatation in the proximal arch. However, longitudinal imaging studies are required to confirm causation.

### Aortic wall shear stress and flow displacement

Association between outflow asymmetry and maximum systolic WSS link the altered haemodynamics to the asymmetric shear stress patterns that may lead to aortic remodelling, shown in Figure 3.^23,43^ Regional increases in aortic WSS have also been correlated with eccentric outflow jet direction for different BAV fusion types.^64–67^ In one study, jet flow impingement was found mainly to elevate the right anterior WSS of the aorta for RL-BAV types and the right posterior WSS for RN-BAV types.^40^ Another study explored the correlation between BAV fusion types and aortopathy phenotypes according to the proposed definitions^68,69^: Type 0: normal aorta; Type 1: dilated aortic root; Type 2: aortic enlargement involving the tubular portion of the AAo; and Type 3: diffuse involvement of the entire ascending aorta and the transverse aortic arch.^29^ The majority of RL-BAV patients (13 out of 15) exhibited Type 2 phenotypes, while RN-BAV patients had a much higher incidence of Type 1 (8 out of 15) and Type 3 (5 out of 15) aortopathies. While flow displacement and high WSS are found to be sensitive predictors of BAV phenotypes with good inter- and intraobserver variability,^27,56,70^ this study was however limited by a lack of age-matched controls.

Given aortic diameter and growth rate are subject to measurement error and are not ideal predictors of acute aortic syndrome^71,72^ WSS may be a better predictive marker.^34^ Statistically significant association between the circumferential component of WSS and local aortic growth rate in recent longitudinal studies suggest that WSS can potentially identify patients at risk for aortic dissection.^34,35^ In another study, however, the percentage area of elevated WSS was greater in BAV patients with faster rates of AAo growth while no association was demonstrated by maximum and mean systolic WSS values.^36^ This would indicate that WSS heat maps are better predictors of faster aortic growth rate.^36^

On the other hand, both high and low WSS have been separately associated with intracranial aneurysmal growth and rupture.^73,74^ These contradictory findings have prompted the suggestion that aneurysm formation may be driven by two different mechanistic pathways. High spatial WSS triggers mural-cell-mediated destructive remodelling, whereas low WSS triggers inflammatory-cell-mediated destructive remodelling when coupled with a high oscillatory shear index, a dimensionless parameter that describes the directional variations in WSS throughout the cardiac cycle.^75–77^ Nonetheless, regional abnormalities of low aortic WSS and high oscillatory shear index were shown not to be concomitant with aortic dilatation regions by 4D flow in patients with BAV, thus opposing the low and oscillatory shear stress pathogenesis theory in BAV-related aortic dilatation.^38^

However, WSS is known to be underestimated due to the limited temporal and spatial resolution of 4D flow and is restricted to measurements at predefined levels.^78,79^ Volumetric WSS methods have since been proposed but have yet to be used in practice.^56^

### Pressure mapping

Aortic valve pressure gradients measured by echocardiography or catheterisation are subject to overestimates due to pressure recovery.^80^ Catheterisation also has the inherent risks associated with invasive procedures.

Pressure drop mapping may be another feasible proxy for AS severity in describing abnormal blood flow.^39^ The maximum pressure gradients at nine pre-defined aortic locations between the left ventricular outflow tract and the descending aorta have been shown to be significantly higher in BAV patients than in TAV controls. Despite good intra- and interobserver variability between pressure drop measurements, variable bias is demonstrated in the scan–rescan assessment depending on measurement location. Pressure drop measurements are difficult to compare with absolute values, such as catheterisation measurement, as they depend on an arbitrarily specified reference point.^39^ The discrete spatiotemporal resolution of 4D flow and signal dephasing from turbulence and complex flow also compromise accuracy.^79,81^

### Flow energetics

Energetic losses due to the altered transvalvular flow dynamics and pressure loss are of particular interest in 4D flow as a potential non-invasive alternative to assessing AS severity with increased accuracy. Moreover, the energetic losses provide a measure of the increased left ventricular load required to overcome this loss, serving as a potential prognostic marker for the degree of left ventricular dilatation and failure.^82^ Kinetic energy can be decomposed in the mean kinetic energy and turbulent kinetic energy components. Viscous dissipation of turbulent kinetic energy into heat is the dominant cause of irreversible pressure loss in the transitionally turbulent flow regime distal to the vena contracta.^83,84^

**Figure 4.**
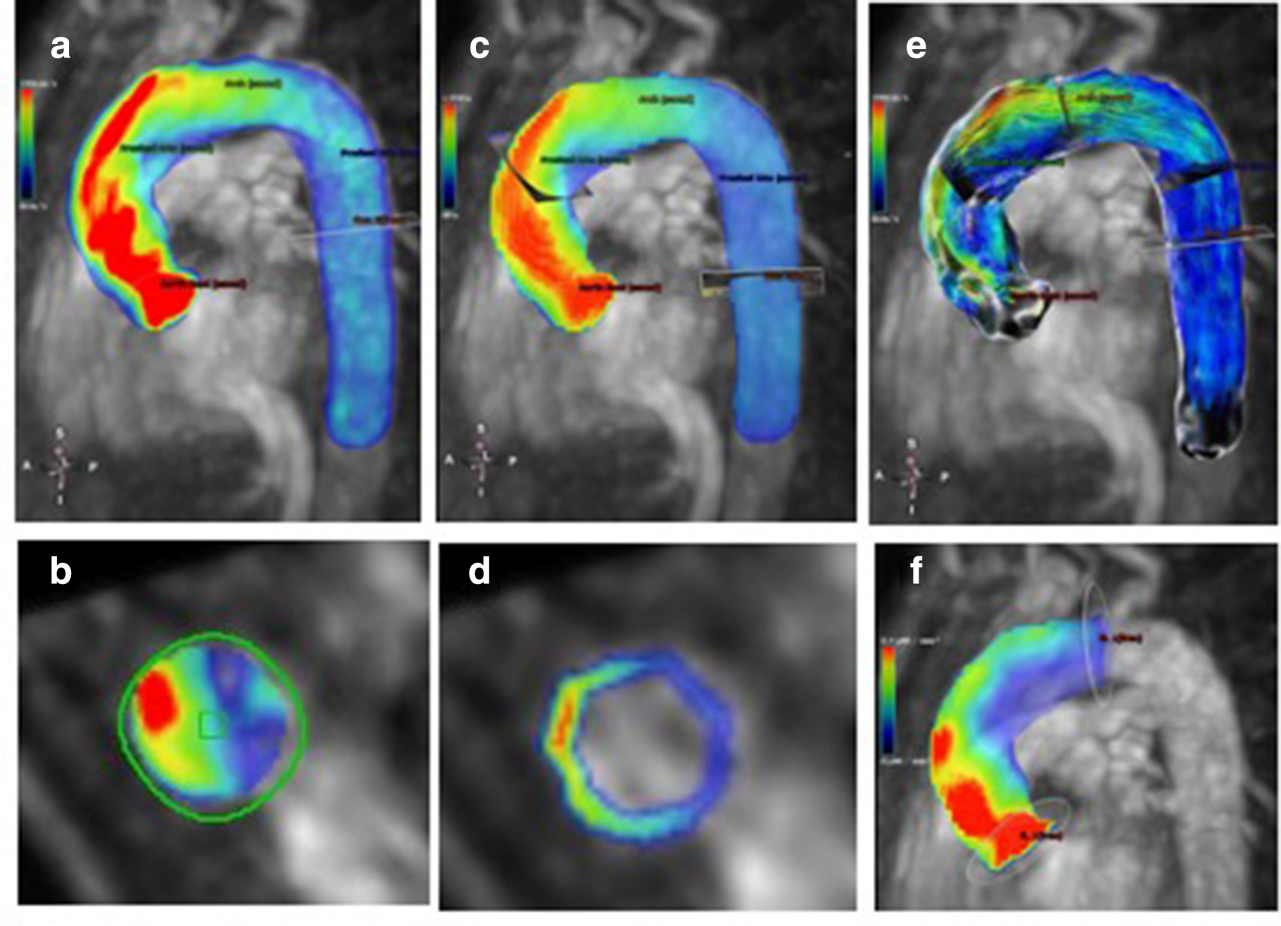
4D flow CMR-based characterization of flow dynamics at peak systole in a 60-year-old male with a bicuspid aortic valve and symptomatic aortic stenosis (NYHA class 3) using CVI42 (Circle Cardiovascular Imaging Inc., Calgary, Canada). (A) Velocity-coded 4D flow CMR reveals an eccentric asymmetric flow jet (indicated by yellow and red areas in heatmap) in the ascending aorta. (B) The flow jet impacts and travels along the right aortic wall at the mid-ascending aorta. (C) Corresponding wall shear stress with regional maxima coinciding with velocity profiles. (D) Wall shear stress at the level of the mid-ascending aorta shows the marked asymmetry due to eccentric flow pattern. (E) Pathlines demonstrate mild helical flow with vortices in the ascending aorta. (F) Energy loss (μW/mm^3^) heat map between two perpendicular planes at the aortic root and arch. Elevated energy loss (red areas) indicates areas of increased viscous energy losses and turbulent flow along right aortic wall.

Feasibility estimates of turbulent kinetic energy losses that quantify regions of turbulent flow have been demonstrated by 4D flow measurements.^83^ Significantly elevated turbulent kinetic energies have been measured in patients with dilated AAo or BAV from a cohort comprising mixed severity AS and valve phenotypes as well as healthy age-matched volunteers.^45^ 4D flow can also quantify viscous energy loss caused by regions of abnormal or complex non-turbulent flow, such as vortex formation or helical flow.^85^ Early studies have indicated near double viscous energy losses between the left ventricular outflow tract and proximal first branch of the aorta in BAV patients with aortic dilatation or AS compared to healthy volunteers, although further studies are required for full qunatification.^86^ Figure 4 illustrates the asymmetry in velocity profiles at peak systole in a 60-year-old BAV patient with symptomatic severe AS. Eccentric flow and WSS coincide with regions of high energy loss, indicating increased turbulence at the right aortic wall in the mid-AAo. 4D flow thus has the sensitivity to not only assess AS severity in BAV patients from measurements of energetic losses, but may also differentiate regions of turbulent and non-turbulent blood flow.

### Aortic stiffness

PWV is a clinical measure of arterial wall stiffness by its inverse relation to distensibility and can be calculated using the Bramwell-Hill equation, assuming changes in the vessel wall thickness are negligible compared to the diameter and that the circulating fluid is incompressible and nonviscous.^87^ Increased aortic wall stiffness can lead to left-ventricular remodelling due to the increased afterload.^87,88^ High correlation between 4D flow-derived aortic PWV with carotid-femoral PWV and left-ventricular mass-to-volume ratio provide some evidence for the validity of the metric to examine aortic stiffness.^89,90^

Dissociation between aortic dilatation and PWV was demonstrated in a study using 2D PC MRI in 63 BAV and 106 TAV patients with AS, with higher AAo dimensions in BAV, but no difference in PWV.^91^ Patients with RN-BAV demonstrated significantly lower aortic stiffness (higher distensibility and lower PWV) compared to RL-BAV, despite a trend towards higher AA area. Conversely, another study with 30 non-dilated and 106 dilated BAV patients, 18 dilated TAV and 44 patients with Marfan’s Syndrome suggested an association between distensibility and regional PWV.^41^ The dilated and non-dilated BAV patients were found to have similar PWV and AAo distensibility, after adjustment for AAo diameter, to healthy controls and dilated TAV patients respectively, while both dilated and non-dilated Marfan’s syndrome patients had significantly increased PWV values. The authors thus inferred that BAV and TAV patients, with and without aortic dilatation, exhibited similar regional aorta mechanical properties. In this study, the PWV in the AAo initially decreased with dilatation until a diameter of 50 mm, beyond which the PWV then increased, suggesting that distensibility and PWV should be considered in context with aorta size. The conflicting results suggest a continuing need for further investigation.

## The future

### Standardised analysis

The feasibility of 4D flow acquisition and analysis methods has resulted in a burgeoning of studies on its utility in characterising AS and its associated aortopathy. The consequence is a heterogeneity in current 4D flow data acquisition and reconstruction protocols and the assessment tools for analysing the large volumes of subsequent data generated. The lack of conformity in post-processing and analysis methods hampers cross-site comparison and limits the accuracy and external validity of numerical values. Limited temporal and spatial resolution further restricts the accuracy of derivative calculations of higher-order parameters such as vorticity and shear stress. Better understanding of these limitations and how to overcome them is required to improve 4D flow data quality and standardise its wide-scale clinical applicability.

### Accelerated imaging

K-space undersampling techniques that exploit spatiotemporal correlations such as k-t BLAST or k-t SENSE,^92^ k-t GRAPPA,^93^ and k-t PCA^94^ already allow for acceleration factors of the order of 5 to 8. Compressed sensing is now being offered as a commercial product that achieves increased acceleration rates by incoherent sampling.^95,96^ Acquisition times are reduced by sampling only a small portion of k-space, done semi-randomly (*e.g.* spiral) to eliminate aliasing artefacts. Transform sparsity and non-linear iterative reconstruction remove the noise-like artefacts, superimposed on the image by incoherent sampling, while retaining as much data consistency achievable. This has opened cardiac MRI to a larger patient group such as those with arrhythmia or those who cannot breath-hold. Previously hampered by relatively long offline reconstruction times, ranging 45–60 min, feasibility studies of compressed sensing combined with direct online reconstruction have demonstrated time saving without loss of data quality.^97^ The higher resolutions at constant scan times potentially afforded by compressed sensing may improve the accuracy and precision of the higher-order metrics.

### Automated post-processing

Visualisation and computation of haemodynamic metrics have been previously impeded by labour-intensive manual segmentation of the aorta.^98^ Automated analysis can potentially reduce analysis time and may enable broader clinical applications. Commercially available software now allows rapid, semi-automated segmentation. However, reliance on user-adjusted thresholding for masking and manually positioned analysis planes has resulted in regional sensitivity of measurements to plane placement and increased partial volume error.^48^ Potential segmentation-free methods have been compared favourably to traditional manual segmentation.^48,99^ Advances in machine learning have also markedly improved automated processing. Deep learning methods, such as convolutional neural networks, have the potential for fully automated aortic segmentation. Multisite studies have achieved subsecond segmentation with similar geometrical reproducibility and accuracy of blood flow measurements to manual analysis across a wide range of patient characteristics, vascular territories and congenital heart diseases.^100,101^ Current studies are limited to parameters that may not be particularly sensitive to haemodynamic differences between the TAV and BAV groups.^102^ Further studies with larger multisite, multivendor training data sets are required and the outcomes compared against gold-standard measurements to determine the accuracy, reproducibility, and observer variability of regional flow dynamics for clinical applications.

## Conclusion

Previously hampered by inherently long scan time and a lack of widely available standardised cardiac MRI protocols and analysis methods, 4D flow is now emerging as an important research tool and a potentially cost-effective and feasible technique in future clinical practice. Aorta 4D flow acquisition and analysis methods can provide non-invasive quantitative assessment of aortic blood flow patterns and haemodynamic parameters within clinically feasible times. Quantification of aortic flow can play an important complementary role in the characterisation and management of patients with BAV and associated aortopathy. However, current studies are limited with heterogeneous populations (with and without AS/AR) and control groups, small numbers and heterogeneity in post-processing and reported haemodynamic parameters. Further studies are needed to firmly establish its role in the diagnosis and management of patients with BAV and its associated aortopathy.
